# Characteristics of Carbonate Formation from Concentrated Seawater Using CO_2_ Chemical Absorption Methodology

**DOI:** 10.3390/ijerph18010120

**Published:** 2020-12-26

**Authors:** Sangwon Park, Yeon-Sik Bong, Chi Wan Jeon

**Affiliations:** 1 Center for Carbon Mineralization, Korea Institute of Geoscience and Mineral Resources (KIGAM), 124 Gwahang-no, Yuseong-gu, Daejeon 34132, Korea; psw1231@kigam.re.kr; 2 Earth and Environmental Analysis Group, Korea Basic Science Institute (KBSI), 162, Yeongudanji-ro, Ochang-eup, Cheongwon-gu, Cheongju-si, Chungcheongbuk-do 28119, Korea; bong_geo@kbsi.re.kr

**Keywords:** CCS, CCU, CO_2_ fixation, CO_2_ conversion, recovery of valuable metals

## Abstract

Carbon capture and storage is a popular CO_2_-reduction technology, and carbon capture and utilization (CCU) technology has been reported frequently over the years. However, CCU has certain disadvantages, including the requirement of high energy consumption processes such as mineral carbonation. In addition, stable metal sources are required to fix CO_2_. This study used concentrated seawater to supply metal ions. In addition, the selected 5 wt % amine solution changed CO_2_ into aqueous CO_2_ to reduce the additional energy required to form the metal carbonate under moderate conditions. As a result, precipitates were formed because of the reaction of carbonate radicals with metal ions in the seawater. These precipitates were analyzed by X-ray diffraction and field-emission scanning electron microscopy, and they were found to mostly consist of CaCO_3_ and NaCl. Furthermore, it was verified that the conversion solution maintained its CO_2_-loading capacity even after the solids and liquid were filtered twice. Therefore, the proposed method permits a substantial reuse of CO_2_ and waste seawater when sufficient metal ions are supplied. Therefore, methods to improve their purity will be developed in future studies.

## 1. Introduction

The emission of CO_2_ as a greenhouse gas (GHG) has been increasing worldwide owing to the use of fossil fuels [1]. According to the Intergovernmental Panel on Climate Change (IPCC), CO_2_ is one of the major GHGs, along with CH_4_, N_2_O, HFC, PFC, and SF_6_ [2,3]. CO_2_ is generated by human activities, such as those in coal-fired power plants and steel and cement industries. Among these sources of emission, coal-fired power plants produce the highest amount of CO_2_. Consequently, several researchers have been exploring methods to reduce CO_2_ emissions from such industries. From the point of view of CO_2_ capture, CO_2_-reduction technologies consist of pre and post-combustion as well as oxy-fuel combustion [4]. Among these methods, carbon capture and storage (CCS) is the most popular post-combustion CO_2_ reduction technology used in Korea [5]. CCS can be applied to industries that are responsible for a large amount of CO_2_ emission such as coal-fired power plants [6]. For example, the Korea Electric Power Research Institute has developed 50 MW pilot plants that aim for the reduction of CO_2_ emission.

However, CCS technology requires additional energy to separate the CO_2_ absorbed in the solvent [7]. In general, the absorbed CO_2_ is separated using heat and/or decompression [8,9]. More than 80% of the energy needed for CCS is used during this process [4]. Furthermore, a storage area is required for the captured CO_2_ [9]. Some countries, including South Korea, do not have sufficient space to store the captured CO_2_ [10]. In addition, even if such space can be secured, the stored CO_2_ is not stable [4]. Generally, as mentioned, the separated CO_2_ is stored in the ocean or underground [11]. Therefore, any changes to those areas, for example, an earthquake or a volcanic eruption, could lead to the stored CO_2_ being re-emitted into the atmosphere [5,12]. For these reasons, carbon storage in solids such as carbonate minerals is crucial [13].

Recently, carbon capture and utilization (CCU) has been reported to reduce and change the emitted CO_2_ into more stable materials. “Mineral carbonation” is a representative CCU technology. First suggested by Sefritz in the 1990s [14], this mechanism facilitates the generation of solid-state CO_2_. As expressed by Equations (1)–(4), 1 mol of bivalent metal ion reacts with 1 mol of aqueous CO_2_ ion as follows: 

Net ionic equation of carbonation system in natural water [15]:

H_2_O + CO_2_ ↔ H_2_CO_3_ (1)

H_2_CO_3_ ↔ HCO_3_^−^ + H^+^ (2)

HCO_3_^−^ ↔ H^+^ + CO_3_^2−^ (3)

M^2+^ + CO_3_^2−^ → MCO_3_↓ (4)

In general, CO_2_ is converted into aqueous CO_2_ in water, in forms such as H_2_CO_3_, HCO_3_^−^, and CO_3_^2−^. Thereafter, aqueous CO_2_ reacts with the metal ions present in the solution. In nature, this process is referred to as weathering and it is a slow reaction [16]. Through this process, the CO_2_ in the atmosphere is stored semi-permanently in solid materials. 

However, the natural carbonation process has certain drawbacks. First, the conversion rate of CO_2_ into metal carbonate is low. To form the metal carbonate, the conversion rate of CO_2_ (into carbonate ions, CO_3_^2−^) must be sufficient to rapidly react with the metal sources. Owing to the low conversion rate, a long residence time is required to form metal carbonates through the natural carbonation process. This means that when this process is used in industries, large reactors are required. Second, a stable metal ion supplement is required. Even if the conversion rate of CO_2_ is sufficient to supply the metal ions, metal carbonates will be formed slowly if the concentration of metal ions is low. According to Park et al. [4,6], most of the converted CO_2_ in an amine solution can be used to form metal carbonates inasmuch as there are enough metal ions. Finally, the process requires high temperature and pressure [12,17]. In general, CO_2_ in the gaseous state requires high energy to react with metal ions which are in the solid state. According to the results of previous studies, a pressure higher than 20 bar and temperature higher than 210 °C are required to create an excited state for each ion [9]. Therefore, a higher conversion rate and stable metal ion supplementation must be achieved under low energy consumption conditions.

The purpose of this study is to explore the possibility of reusing wastewater by applying the CCU methodology. Generally, concentrated seawater (desalination seawater) has a high concentration of NaCl and metal ions. Therefore, it could be problematic to release it in the environment; however, its use could help solve environmental problems. To improve the conversion rate of CO_2_, monoethanolamine (MEA), di-ethanolamine (DEA), and methyl-diethanolamine (MDEA) were selected. In our previous studies, amine solutions facilitated an increase in the rate of CO_2_ conversion [4,5,6,16]. Various amine solutions such as MEA, di-ethanolamine (DEA), and methyl-di-ethanolamine (MDEA) have been used before [4,9,16]. Further, concentrated seawater is used to supply stable metal ions [18]. As is widely known, various positive ions (such as Ca^2+^, Mg^2+^, and Na^+^) are dissolved in seawater. These positive ions react well with aqueous CO_2_. Thus, solid metal carbonates (e.g., MgCO_3_, CaCO_3_, etc.,) are formed. Therefore, in this study, it was assumed that, facilitated by the amine solution and concentrated seawater, CO_2_ would rapidly change into metal carbonates. Based on these assumptions, the possibility of recovering valuable metals from seawater was combined with the potential for CO_2_ reduction.

## 2. Theory and Assumptions

### 2.1. CO_2_ Conversion Using Amine Solution

Amine solutions can aid in improving the CO_2_ conversion rate. Generally, an amine solution is Amine solutions can aid in improving the CO_2_ conversion rate. Generally, an amine solution is used as a solvent in the CO_2_ capture process. According to Hook [7], the CO_2_ absorbed in an amine solution changes into aqueous CO_2_ in forms such as carbamate, bicarbonate, and carbonate. In the CO_2_ absorption process, aqueous CO_2_ is indicated by the zwitterion state. The pH of the amine solution varies continuously as CO_2_ is absorbed. The chemical reactions of the absorbed CO_2_ in the amine solution are represented by Equations (5)–(9). The reactions depend on the changes in pH. This means that the pH values of the amine solutions increase and as they become more alkaline the absorbed CO_2_ decreases. The composition of aqueous CO_2_ varies based on these reactions. On the other hand, absorbed CO_2_ can convert to metal carbonate when metal ions are supplied [6]. Thus, the amine solution is changed into a free amine state to absorb CO_2_ again. For this technology to be applicable, it should be capable of not only separating the absorbed CO_2_ but also recovering the valuable metals easily. 

The dominant reactions of CO_2_ with MEA [4,6,9] can be described as follows:

RNH_2_ + H_2_O ↔ RNH_3_^+^ + OH^−^ (5)

OH^−^ + CO_2_ ↔ HCO_3_^−^ (6)

2RNH_2_ + CO_2_ ↔ RNH_3_^+^ + RNHCOO^−^ (7)

RNHCOO^−^ + H_2_O ↔ RNH_2_ (free amine) + HCO_3_^−^ (8)

RNH_2_ + CO_2_ + H_2_O ↔ RNH_3_^+^ + HCO_3_^−^ (9)

As expressed in Equations (1)–(4) and (5)–(9), the absorbed CO_2_ is converted into aqueous CO_2_ in forms such as H_2_CO_3_, HCO_3_^−^, and CO_3_^2−^. These aqueous forms of CO_2_ react with the artificial metal ions supplied as follows: 

Examples of metal carbonate formation: 

Carbamate:

RNHCO_2_^−^RNH_3_^+^ + 2H_2_O + M^2+^ ↔ 2RNH_3_^+^ + H_2_O + MCO_3_↓ (10)

Bicarbonate:

RNH_3_^+^HCO_3_^−^ + RNH_2_ + H_2_O + M^2+^ ↔ 2RNH_3_^+^ + H_2_O + MCO_3_↓ (11)

Carbonate:

2RNH_3_^+^ + CO_3_^2−^ +H_2_O + M^2+^ ↔ 2RNH_3_^+^ + H_2_O + MCO_3_↓ (12)

The CO_2_ absorbed in the amine solution is indicated by the zwitterion state [Equations (10)–(12)]. These ions lose their electrons/negative charge (in this case, aqueous CO_2_) because of the metal sources. The bonding energy of the metal carbonate is higher than that of the aqueous CO_2_ maintained using the amine solution. Therefore, aqueous CO_2_ easily changes into metal carbonates. Furthermore, unlike the CO_2_ separation process implemented in the CCS technology, this process does not use additional energy to produce a metal carbonate. Therefore, the recommended processes could lead to a novel CO_2_ removal methodology and recover valuable metal ions from a multicomponent metal ion solution, which was concentrated seawater in this case. The role of concentrated seawater is explained in the following section.

### 2.2. CO_2_ Mineralization with Concentrated Seawater

For the implementation of the CO_2_ mineralization methodology, a source of stable metal ions is required. Earlier studies have employed artificial metal ion sources such as Ca(OH)_2_, BaCl_2_, and CaCl_2_ [6,9,16]. The authors used simulated metal solutions with a metal ion concentration of 20 wt % with balanced water. That is, the concentration of the supplied metal ion was higher than the amount of absorbed CO_2_ because the concentration of the latter was approximately 0.2–0.5 mol in the amine solution [4,9,16,19]. Therefore, most of the absorbed CO_2_ in the amine solution was converted into metal carbonate. For this reason, we selected concentrated seawater, which, according to Dickson and Goyet [18], is widely known to dissolve various metal ions and thus, is a suitable source of metal ions. Specifically, Na, Mg, and Ca are the major dissolved metals of seawater at a salinity of 35 [20]. Thus, the dissolved metal ions can be recovered using aqueous CO_2_. Although the CO_2_ mineralization method can easily be used to form a metal carbonate, in our study, the general CO_2_ mineralization method could not recover the metal ions from natural seawater because the concentration of dissolved metal ions was low. However, certain processes such as salt refining and desalination produce concentrated seawater. According to Wang and Li, concentrated seawater has the potential to supply stable metal ions and form metal carbonates [21]. Equations (13) and (14) prove that concentrated seawater can produce metal carbonates: 

Formation of metal carbonate with aqueous CO_2_ using concentrated seawater

Carbamate:

RNHCO_2_^−^RNH_3_^+^ + 2H_2_O + M^2+^ + NaCl → 2RNH_3_^+^ + H_2_O + MCO_3_↓ + NaCl↓ (13)

Bicarbonate:

RNH_3_^+^HCO_3_^−^ + RNH_2_ + H_2_O + M^2+^ + NaCl → 2RNH_3_^+^ + H_2_O + MCO_3_↓ + NaCl↓ (14)

Carbonate:

2RNH_3_^+^ + CO_3_^2−^ +H_2_O + M^2+^ + NaCl → 2RNH_3_^+^ + H_2_O + MCO_3_↓ + NaCl↓ (15)

However, concentrated seawater is expected to form two types of major precipitates, NaCl and CaCO_3_ [Equations (13)–(15)], because its major component is sodium. The solubility of NaCl in the solution decreases because CO_2_ is absorbed into a solution of amine and concentrated seawater. Simultaneously, a CaCO_3_ precipitate is formed with NaCl. According to Sun et al. [22], the formation of MgCO_3_ is an exothermic reaction. This means that additional heat is required to produce MgCO_3_ from the concentrated seawater, although the Mg ion is one of the major components of concentrated seawater. For this reason, the formation of MgCO_3_ was not observed under the conditions of this study. The case of CaCO_3_ is different. According to Romão et al. [23], CaCO_3_ was formed to absorb heat because it is an endothermic reaction. In this study, we assume that MgCO_3_ does not require additional energy to produce a metal carbonate. Therefore, concentrated seawater and amine solution could produce NaCl and CaCO_3_ first. Considering the metal ion concentration and CO_2_ mineralization reactions, concentrated seawater is an adequate metal supplement resource.

Furthermore, the amine solution used can reabsorb the emitted CO_2_ under identical experimental conditions. As expressed by Equations (13)–(15), the amine solution loses the absorbed CO_2_.

## 3. Materials and Methods

**Figure 1:**
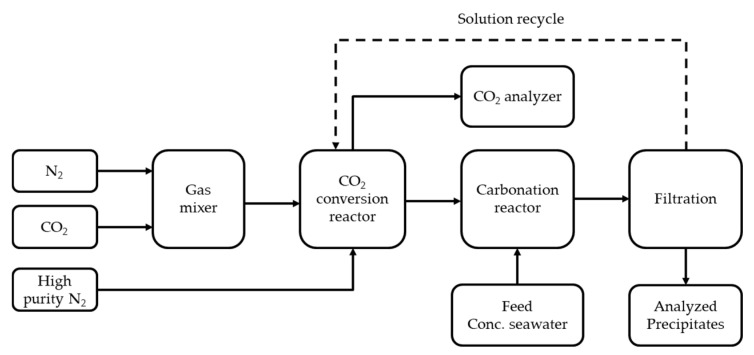
Schematic diagram and apparatus for experiment.

The flow schematic of the experiment is depicted in Figure 1. To verify the results of our study, we assumed that the concentration of CO_2_ was 15 vol%. A mass flow controller was used to achieve a balance between N_2_ gas (purity: 99%) and CO_2_ gas (purity: 99%). We used a CO_2_ analyzer (Sensor Lonic Co. Ltd., Korea) to regulate the gas flow rate to 1880 mL/min and 300 mL/min for N_2_ and CO_2_, respectively. Before the experiment, all reactors were purged with N_2_ (purity: 99.999%) to remove any trace gases. Finally, we performed an experiment in which none of the residue gases in any of the reactors were detected by the analyzer. The simulated gases (N_2_ and CO_2_) were mixed in a saturated reactor before they were introduced into the CO_2_ conversion reactor made of Pyrex glass. The saturated reactor was filled with water, and its temperature was maintained at 30 °C. The temperature was controlled using a thermal water bath. From the saturated reactor, the simulated gas flowed into the conversion reactor after passing through a Teflon tube, where it was dispersed using a bubble diffuser. The conversion reactor was filled with 5 wt % MEA balanced water. The selected amine with purities higher than 99% was purchased from Sigma Aldrich Co. Ltd. The conversion solution volume was 400 mL. The venting gas flowed continuously into the CO_2_ analyzer at 30 s intervals. We assumed that the CO_2_ conversion solution was saturated by CO_2_ when its concentration in the venting gas was 15 vol%, as indicated by a CO_2_ analyzer. The venting gas was passed through the condenser, which was maintained at a temperature of less than −5 °C to maintain the concentration of the conversion solution by preventing vapor loss. Throughout these processes, the MEA concentration was kept constant at 5 wt %. We added concentrated seawater to the CO_2_-saturated conversion solution when the first conversion reaction was completed. To recover the metal carbonate, concentrated seawater was used (Table 1), because it matched the total CO_2_-loading values and the metal ion rate. In a previous study, Park et al. [16,24] reported that most of the converted CO_2_ could form metal carbonates when the concentration of metal ions was adequate for the reaction. Thereafter, the CO_2_-saturated solution (amine in this study) was mixed with concentrated seawater. Next, we used a magnetic stirrer at 200 rpm for 24 h. The precipitate was separated using filter paper with a pore size of 0.7 μm. The separated precipitate was dried in an oven at 105 °C for 24 h. The dried material was washed with water to remove soluble components such as NaCl. Finally, the residue solid was dried again in an oven. The dried separated solids were analyzed using X-ray diffraction (XRD) to determine the composition at each step (first and second dried samples). Finally, we used a desorption process to check the amount of residual CO_2_ in the solution (separated liquid). Only N_2_ gas at 70 °C was used in the desorption experiment, and its flow rate was maintained at 1880 mL/min until the CO_2_ analyzer indicated 0 vol%. The retention time was maintained for 30 min after it reached 0% according to the CO_2_ analyzer. Finally, we performed the same experiment again to confirm that the separated conversion solution could be reused twice under identical experimental conditions.

## 4. Results and Discussion

### 4.1. CO_2_ Loading and Conversion

**Figure 2:**
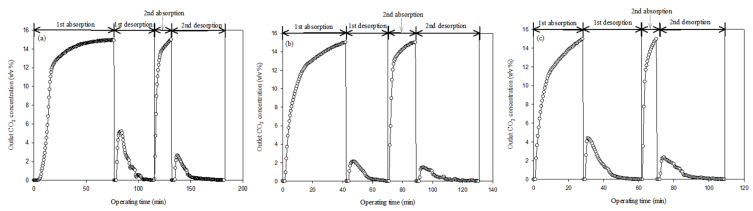
Results of CO_2_ loading at 5 wt % for each amine by (re-)absorption and (re-)desorption. First desorption and second absorption experiments as checked by the residual CO_2_ remaining after reaction with metal ions in concentrated seawater: (**a**) monoethanolamine (MEA) solution, (**b**) di-ethanolamine (DEA) solution, and (**c**) methyl-diethanolamine (MDEA) solution.

Figure 2a–c present the CO_2_-loading results of the three selected types of 5 wt % amine solutions. Considering the previously obtained results, the 5 wt % MEA solution was better than the 30 wt % MEA solution for operating the general process. A higher-concentration MEA solution required a longer reaction time than a lower-concentration one. This means that the operation time could be reduced by using a lower-concentration MEA solution [4,6,16]. As mentioned above, we used absorption and desorption in experiments twice under identical conditions. We used a 5 wt % amine conversion solution because it could adapt to the absorbed CO_2_ and metal ion concentrations. According to Park et al. [9], a high concentration of amines is required along with a similar concentration of metal ions to produce a precipitate. Earlier, we explained the metal ion composition of seawater as proposed by the carbon mineralization theory. The amount of converted CO_2_ in the conversion solution was higher than the amount of CO_2_ used in the formation of metal carbonate. Therefore, the residual CO_2_ in the solution can be re-emitted into the atmosphere or concentrate if it does not react with the metal ions. Consequently, a low concentration of the conversion solution was considered advantageous. Rapid CO_2_ conversion was required to provide the metal ions needed to produce the metal carbonate, and the conversion solution could be rapidly reused because the total CO_2_-loading capacity was lower than that of the high-concentration solution. The low concentration of the added metal ions could also be applied to the low-concentration conversion solution. This result indicated that the carbonate precipitate and conversion solution reuse cycle time would be reduced. According to Park et al. [4], a 5 wt % amine solution had a lower CO_2_-loading capacity than a 30 wt % amine solution. Therefore, experiments were conducted using a 5 wt % amine solution to reduce the reaction time. We expect that this will help reduce the process scale.

As illustrated in Figure 2, we were able to determine the amount of converted CO_2_. The first and second steps were absorption and desorption, respectively. These results were similar to those obtained by other researchers. According to Park et al. [6,24], the general CO_2_-loading value in the absorbent increased when the emitted CO_2_ flowed into the absorbent. The desorption process also re-emitted the absorbed CO_2_ into the atmosphere by heat (70 °C), and only N_2_ gas was used. Furthermore, Kang et al. [19] reported that the CO_2_-loading capacities of the 5 wt % amine solution for MEA, DEA, and MDEA were 0.1545, 0.0955, and 0.0761 mol of CO_2_, respectively. They reported that the CO_2_-loading capacities decreased when identical experimental conditions were used to check for changes (MEA 0.1357, DEA 0.0911, and MDEA 0.0848 mol of CO_2_) [19]. This result indicates that the added artificial metal sources could be attributed to a decrease in the CO_2_-loading capacity because CaO was used to fix the converted CO_2_ in the amine solution. According to Park et al. [24], this was caused by zwitterion complexes as they produce various types of aqueous CO_2_ (HCO_3_^−^, H_2_CO_3_, CO_3_^2−^). In particular, the CO_2_-loading capacity of the 30 wt % amine solution was not three times greater than that of the 5 wt % solution because it was caused by different amine molecular weights [19]. Considering the results obtained by other researchers, the CO_2_ conversion results (absorption) obtained in this study also indicated a tendency similar to that of typical amines (Table 2). 

However, the amount of emitted CO_2_ in the desorption process was not the same as the amount of absorbed CO_2_ in the typical desorption process reported by other researchers. This result was attributed to the presence of other positive and negative ions in the seawater [20], generated by the absorbed CO_2_ (aqueous CO_2_), that reacted with the metal sources present in the seawater. As previously stated, various positive ions are dissolved in seawater. Among these, sodium chloride (NaCl) was the major component. We were not sure whether the highly concentrated NaCl would have a decisive effect on the CO_2_ loading. In addition, we assumed that the formation of the metal carbonate would incorporate aqueous CO_2_ with metal sources from the seawater. As mentioned earlier, we used artificial concentrated seawater. Therefore, several ions were involved in the reaction, and the concentration of NaCl in our study was greater than that in normal seawater. Therefore, we hypothesized that this would cause an interference response. Hence, the reaction generated a precipitate that included aqueous CO_2_ in forms such as carbamate, bicarbonate, or carbonate, and similar curves were observed for each amine. In the case of MEA, there was a larger amount of converted aqueous CO_2_ compared to the cases of other amines because the CO_2_-loading time was longer. 

In addition, the reabsorption test indicated a relative decrease in the CO_2_-loading time (Figure 2). The absorption time for MDEA was shorter than that for DEA. A small amount of amine was removed from the precipitate and liquid during the separation process. However, we believe that this technique has the potential to decrease the CO_2_ loading owing to the presence of other ions in the seawater when we consider the results of a previous study by Park et al. [4,9]. They assumed that the decrease in CO_2_ loading was caused by the separation process. The results of their second step decreased by approximately 0.02 mol-CO_2_/mol of amine. From this point of view, the large number of ions in concentrated seawater was detrimental to the reuse of the conversion solution. Kang et al. [19] indicated that the amount of absorbed CO_2_ in the MEA solution was higher than that in the DEA and MDEA solutions under identical absorption time conditions. Regarding the conversion of CO_2_, we assume that the CO_2_ loading time is not significant because we also assume that there is a possibility of CO_2_ fixation when concentrated seawater is used. Other researchers have claimed that metal ion concentration is more significant when compared with the CO_2_-loading capacity [5,16,24]. This result indicates that the amount of metal carbonate formed depended on the amount of added metal ions to fix the CO_2_, and this was confirmed by our results. Therefore, the desorption curves obtained in our study were different from those obtained in other studies when we used the desorption process to check the amount of residual CO_2_ in the amine solution. Further details are presented in the following section. 

### 4.2. Verification of Precipitate Formation

We conducted a metal carbonate (precipitation) experiment after the CO_2_ conversion step in the amine solution was. From the results of this experiment, we confirmed that the solution rapidly changed to opaque when the concentrated seawater was added to the CO_2_-saturated amine solution. We assumed that this was due to the formation of solids (metal precipitate) because the concentrated seawater was perfectly transparent. To verify the formation of precipitates, the opaque solution was separated into solids and liquids using a vacuum pump. The separated precipitates were measured by weight at each step of the experiment after the first and second reactions. In the first precipitation experiment, the weights of MEA, DEA, and MDEA precipitates were 25.8, 15.4, and 16.2 g, respectively (Table 3). In the second, their weights were 132.4, 109.1, and 89.3 g, respectively (Table 3). Thus, it was concluded that the concentrated seawater provided enough positive ions to rapidly cause CO_2_ precipitation.

We believe that these results have significant implications. Considering the weights, more precipitate was formed in the second step than in the first one, because the second experiment used the same concentrated seawater after the first experiment. That is, the second experiment used the separated solution from the first experiment. Therefore, some of the unreacted ions in the separated solution (first experiment) were assumed to participate in the formation of the second precipitate. From Table 1, Table 2 and Table 3, we were able to calculate the amount of precipitate theoretically. However, the actual amount generated differed from the theoretical calculation. Our previous studies used only singular chemicals such as Ca, Mg, and Ba to produce metal carbonates [4,5,9,16,24]. These cases were more suitable for calculating the expected precipitates than the present case. Previous studies considered only one reaction, in which the amount of CO_2_ dissolved in the solution and the singular chemical concentration were considered. However, in the present study, we must consider several variables because of the multi-ion solution used. A more detailed explanation of this phenomenon and its implications are discussed in Section 4.4.

XRD was used to verify the composition of the precipitate, and NaCl was found to be the major component. The used seawater had a higher concentration than natural seawater. Thus, the solubility of the total number of ions exceeded this limit.

**Figure 3:**
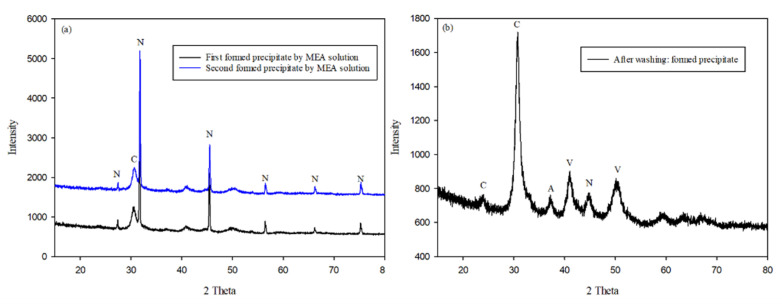
XRD results for precipitate formed in 5 wt % MEA solution with simulated seawater: (**a**) unwashed precipitate and (**b**) washed precipitate. (N: NaCl, C: calcite, V: vaterite, A: aragonite).

**Figure 4:**
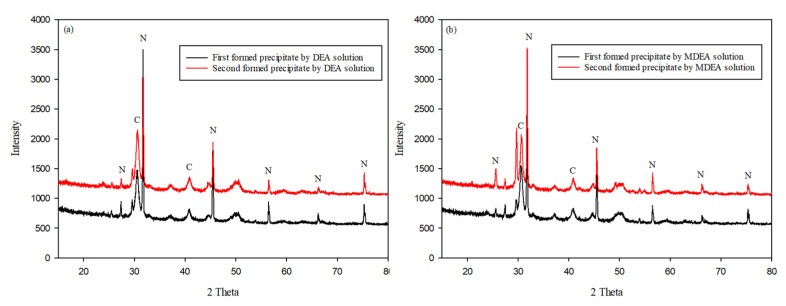
Verification of the precipitates in 5 wt % amines by XRD, except for MEA solution. The formed precipitate was not washed in water. The results were like those in Figure 3a, because the formed sodium chloride concentration was higher than that of the other precipitates: (**a**) aqueous CO_2_ with 5 wt % DEA solution with concentrated seawater and (**b**) aqueous CO_2_ with 5 wt % MDEA solution with concentrated seawater. (N: NaCl, C: calcite, V: vaterite, A: aragonite).

**Figure 5:**
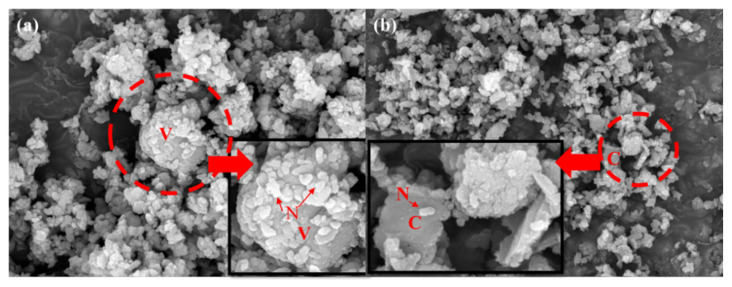
SEM image of carbonate in 5 wt % MEA solution. The formed CaCO_3_ was not visible, because of the sodium chloride (**a**). However, it appears differently in (**b**) because of washing the NaCl. (**b**) shows the CaCO_3_: (**a**) correspond to not washed carbonate and (**b**) to washed carbonate. (N: NaCl, C: calcite, V: vaterite).

Figure 3a and Figure 4a,b, present similar results. The other precipitates could not be distinguished well because the concentration of NaCl in the major precipitate was very high. Ca^2+^ metal ions existed in the seawater, but their concentrations were relatively low when compared with those of NaCl. However, a certain amount of CaCO_3_ was formed on the reaction of aqueous CO_2_ and dissolved Ca ions in the seawater. We considered how this result could be verified with the precipitate. As previously stated, the aim of this study is to verify the potential of applying seawater and CO_2_ conversion solutions. We focused on removing the major precipitate, NaCl, which dissolves easily in water. Therefore, we dissolved the precipitate in water to examine the metal carbonates. An insoluble solid is formed by the metal carbonates depicted in Figure 3b and Figure 5. Most of the precipitates consisted of CaCO_3_, for example, vaterite, aragonite, and calcite. Although there were several positive ions in seawater, the major carbonate was CaCO_3_ because its formation requires heat. This means that the formation of CaCO_3_ is an endothermic reaction [25,26]. In addition, the amount of MgCO_3_ formed is generally lower than that of CaCO_3_ because the enthalpy of CaCO_3_ is lower than that of MgCO_3_ [27]. However, the results obtained in this study indicate otherwise. As mentioned previously, additional heat is required to form MgCO_3_. According to Fricker et al. [28], the advantage of high temperature is that MgCO_3_ can be produced. On the other hand, the formation of CaCO_3_ is an exothermic reaction [26]. Furthermore, MgCO_3_ does not form well when Ca and Mg ions are mixed. In particular, the concentration of Mg ions was higher than that of Ca ions [29]. Considering the results obtained by other researchers, we can conclude that most of the precipitate was CaCO_3_. For this reason, the formation of MgCO_3_ was not observed under the conditions of this study.

The calculation of the concentration of calcium ions in the concentrated seawater was done as follows:

(16) $${\mathrm{Ca}}^{2+}\mathrm{concentration}\;\mathrm{in}\;\mathrm{seawater}\;\left(\frac{\mathrm{mg}}{l}\right)\times \;\frac{\mathrm{mol}}{40\;g}\;\times \;\frac{g}{1000\;\mathrm{mg}}\;\times 1\;L=\;0.6315\;{\mathrm{mol}\;\mathrm{of}\;\mathrm{Ca}}^{2+}$$

The formation of metal carbonate using seawater at 25 °C can be expressed as follows:

Sodium chloride:

Na^+^ + Cl^−^ → NaCl Δ*G*° = −384 kJ/mol (17)

Calcium carbonate:

Ca^2+^ + CO_3_^2−^ → CaCO_3_ Δ*G*° = −1128 kJ/mol (18)

Magnesium carbonate:

Mg^2+^ + CO_3_^2−^ → MgCO_3_ Δ*G*° = −1012 kJ/mol (19)

Potassium carbonate:

2K^+^ + CO_3_^2−^ → K_2_CO_3_ Δ*G*° = −1064 kJ/mol (20)

This can be explained by examining the thermodynamic properties. The Gibb’s free energy (Δ*G*°) for CaCO_3_ [−1128 kJ/mol] was lower than that of other compounds. Equations (16)–(20) show the Δ*G*° values of the other precipitate components of the representative metal ions in seawater. The precipitate was dissolved in water, and a small amount of NaCl was present in the residue. However, we believe that our proposed method has potential for CO_2_ fixation with seawater because the amount of CO_2_ converted in the selected amines was very small (Table 2), and the concentration of the added calcium ions in the seawater was 0.6315 mol (Equation (16)). Therefore, most of the converted CO_2_ in the amine solution formed a metal carbonate. In particular, the amount of converted CO_2_ was lower than that of the added calcium ions. Therefore, a large amount of the second precipitate was formed, especially NaCl. This indicated that the residual metal ions were not dissolved until the second absorption experiment. Therefore, they could react directly with the absorbed CO_2_. Consequently, the total amount of the second precipitate was higher than that of the first precipitates. Therefore, the second CO_2_ absorption capacity also decreased because the CO_2_ analyzer checked the gas vented after it was passed through the absorption reactor. Considering Equations (17)–(20), the value of Δ*G*° for calcium carbonate was lower than that for the other compounds. This result indicated that calcium carbonate formed more easily than other metal carbonates when aqueous CO_2_ reacted with the metal ions.

### 4.3. Characteristics of Precipitates and Its Morphology

**Figure 6:**
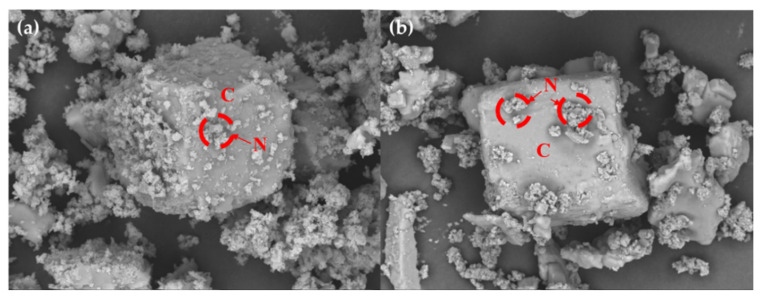
SEM image of carbonate in 5 wt % DEA and MDEA solution: (**a**) DEA solution and (**b**) MDEA solution. (N: NaCl, C: calcite).

**Figure 7:**
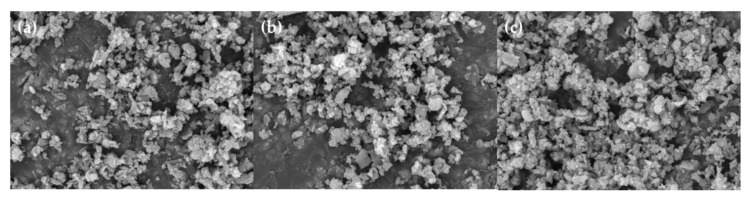
SEM image of first carbonate in 5 wt % MEA, DEA, and MDEA solutions: (**a**) formed precipitates in MEA solution, (**b**) formed precipitates DEA solution, and (**c**) formed precipitates MDEA solution.

In this study, we explored the possibility of recovering useful materials and reducing CO_2_ using concentrated seawater. Figure 5, Figure 6 and Figure 7 present the results of scanning electron microscopy (FE-SEM). As depicted in Figure 5a,b, most of the formed NaCl was removed. Figure 6 shows the CaCO_3_ formed when DEA and MDEA solutions are used. The results obtained are close to those obtained in the MEA case. This means that soluble materials can easily be removed and reused in various industries. Although with concentrated seawater we did not use the NaCl dissolution step for the DEA and MDEA, we could assume that similar results would be obtained for NaCl dissolved in water. Consequently, we confirmed that the fixation of CO_2_ with seawater is possible. In addition, from an environmental point of view, concentrated seawater can be used after desalination. However, this phenomenon needs to be thoroughly investigated. The major limitation was the purity of the precipitate because the major components were CaCO_3_ and NaCl. Through the first precipitates (CaCO_3_, NaCl) formation, trace metal ions could remain in the solution, such as Li, Nd, and In. Moreover, the precipitate has potential value for reuse as a construction material or in DeSO*x* processes; this potential should be explored further [30,31]. However, the purity of the precipitate was too low for effective reuse and needs to be improved for use in other industries [13]. To improve the purity, additional research is needed.

### 4.4. Relation of Ionic Strength and Precipitates at Each Step

To analyze the mechanism of formation of precipitates, we assumed that pH changes and ionic strength (*I*) were significant factors. Therefore, we checked the pH changes when CO_2_ was dissolved in each amine solution. Table 4 presents the pH values at each step of the experiment. The initial pH values for the first absorption were: 11.48 for MEA, 10.92 for DEA, and 11.05 for MDEA. These values decreased to 7.84, 7.82, and 7.68, respectively, when each of the amine solutions was saturated by CO_2_. These values increased when concentrated seawater was added to each solution and desorption was carried out. Finally, the pH values measured in the second experiment were lower than those measured in the first step. Nevertheless, these values decreased, and the final pH values were close to those measured in the first conversion and desorption experiments. In our study, we assumed that the variation in pH did not have a significant effect because the amounts of CaCO_3_ measured in the first and second precipitates were similar. In our previous studies, pH values were not a significant factor for generating metal carbonate [16]. We used amine solutions to convert aqueous CO_2_ into metal ions earlier as well. Thus, the pH values varied continuously as the experimental conditions changed. Additionally, the amounts of precipitate obtained at each step were similar. Consequently, we attempted to identify the factors affecting the CO_2_ fixation with metal ions.

We expected that ionic strength would be a significant factor in determining the precipitate rather than the pH values. Nevertheless, pH values are related to ionic strength, and concentrated seawater has high ionic strength. 

Ionic strength (*I*) = $\frac{1}{2}\sum_{i}c_{i}z_{i}{}^{2}$,

where *c_i_* is the molar concentration of ion *i* (M, mol/L) and *z_i_* is the charge on that ion.

As presented in Table 4, the ionic strength of the first CO_2_ conversion and precipitation experiment was lower than that of the second experiment, in which the separated solution obtained from the first experiment was used. Thus, the second experiment yielded high ionic strength values. Therefore, the significant factor is the overall ionic strength value. In both experiments, the ionic strength was approximately doubled. However, the total of precipitates increased at least by a factor of six (Table 3). Generally, ionic strength is well-known to be related to ion activity. Furthermore, ion activity is affected by ionic strength. In general, ion activity is equal to 1, but it could decrease by more than 1 when the ionic strength increased. In this study, the ionic strength was found to be high in all experiments. Thus, we conclude that the ion activity is lower than 1. This implies that the degree of dissociation dramatically increases because the various metal ions are dissolved in concentrated seawater. For these reasons, the precipitates obtained in the second experiment decreased when compared with those in the first experiment. Table 3 indicates that the amount of CaCO_3_ formed in each solution in the first experiment was higher than that in the second step. According to Lee et al. [32], the temperature and ionic strength are closely related to the saturation. In their study, the precipitate formed faster and with a higher degree of saturation [33]. However, in our case, the amount of NaCl formed in the second experiment was larger than that in the first experiment. We assumed that this was caused by its solubility—1 g/2.8 mL in water [34]. Considering its solubility and degree of dissociation, it can be inferred that NaCl could not be formed under our experimental conditions. However, it was formed at each step and for each solution. In particular, a very large amount was formed in the second experiment. This implies that the solubility of NaCl is significantly predominant over the physicochemical reaction; nevertheless, the chemical reaction (degree of dissociation) is activated by increasing the general ion activity. Consequently, we conclude that solubility becomes significant when a multicomponent solution is used to generate a metal carbonate or to separate some target material.

## 5. Conclusions

The aim of this study was to convert the CO_2_ emitted from industries into metal carbonate using concentrated seawater by applying the rapid carbonate method. Using these processes, waste seawater can be recycled to recover metal carbonates and fix CO_2_. To accomplish our objective, we selected certain amines to rapidly convert CO_2_ into aqueous CO_2_, and concentrated seawater to supply the positive ions. The selected amines were sufficiently qualified for the purpose of improving the CO_2_ conversion rate. From our experiments we concluded that low-energy consumption methods (under mild conditions, 30 °C, 1 bar) demonstrated reasonable potential for CO_2_ removal, fixation, and utilization, with concurrent metal recovery from concentrated seawater. Consequently, in the future, we plan to improve the selectivity during separation considering real waste-concentrated seawater. We expect that future research will lead to the production of high-purity carbonates that can be used in chemical industries.

## Figures and Tables

**Table 1:** Components of concentrated seawater.

Ions	Natural Seawater Conc. (mg L^−1^)	Concentrated Seawater Conc. (mg L^−1^)
Chloride	19,336	116,016
Sodium	10,752	64,512
Magnesium	1317	7902
Calcium	421	2526
Potassium	442	2652
Total ionic strength (*I*)	0.642	3.855

**Table 2:** Results of CO_2_ loading of amines selected for the present study.

	^a^ First Absorption (mol of CO_2_)	^b^ Second Absorption (mol of CO_2_)	^c^ Difference Values (mol of CO_2_)
MEA	0.1988	0.0532	−0.1456
DEA	0.1089	0.0409	−0.068
MDEA	0.0821	0.0289	−0.0532

This table shows the decreased CO_2_ loading capacity when we used the (simulated) concentrated seawater. ^c^ Difference values (mol of CO_2_) = ^b^ Second absorption (mol of CO_2_) − ^a^ First absorption (mol of CO_2_).

**Table 3:** Amount of precipitates formed in each step and for each solution.

Formed Precipitates	MEA				DEA				MDEA			
	Theo. Cal.		Act. Gen.		Theo. Cal		Act. Gen		Theo. Cal		Act. Gen	
step	1st	2nd	1st	2nd	1st	2nd	1st	2nd	1st	2nd	1st	2nd
NaCl	64.51	124.97	20.7	127.6	64.51	126.99	10.4	104.3	64.51	126.75	11.6	84.6
CaCO_3_	1.26	1.5	5.1	4.8	1.26	1.52	5	4.8	1.26	1.61	4.6	4.7
MgCO_3_	3.95	7.9	-	-	3.95	7.9			3.95	7.9		
Total	69.72	134.4	25.8	132.4	69.72	136.41	15.4	109.1	69.72	136.26	16.2	89.3
Eff. (%)			80.6	63.9			79.3	63			72.9	58.6

Unit of formed precipitates = g. 1st Theo. Cal.: (2526 mol-Ca conc. in concentrated seawater/L × 1 L/1000 mL)/100.0869 g/mol. 2nd Theo. Cal.: (X mol-residue Ca conc. after 1st reaction/L × 1 L/1000mL mL)/100.0869 g/mol. Efficiency (%): (Actual generation (Act. Gen.)/Theoretical calculation (Theo. Cal.)) × 100.

**Table 4:** pH changes at each step of the experiment for each amine solution.

Solution	1st Absorption		1st Conversion and Desorption	2nd Absorption		2nd Conversion and Desorption
	Before	After		Before	After	
MEA	11.48	7.84	7.74	8.48	6.8	7.38
DEA	10.92	7.72	7.49	8.1	6.61	7.38
MDEA	11.05	7.68	7.81	7.83	6.62	7.35
